# A broadband and low cross polarization antenna with a balun of microstrip line coupling to slot line

**DOI:** 10.1371/journal.pone.0194181

**Published:** 2018-03-15

**Authors:** Kai Sun, Deqiang Yang, Sihao Liu, Tianming Yang

**Affiliations:** 1 School of Electronic Engineering, University of Electronic Science and Technology of China, Chengdu, Sichuan, China; 2 School of Energy Science and Engineering, University of Electronic Science and Technology of China, Chengdu, Sichuan, China; Beijing University of Posts and Telecommunications, CHINA

## Abstract

In this paper, a wide-band low cross polarization antenna with a structure of microstrip line coupling to slot line as the balun is proposed. The radiation part of the antenna is fed by two pairs of parallel transmission line via a transition from a slot line which is coupled by a microstrip line. Because it is fed by parallel transmission lines, which is balanced-fed structure, the antenna can achieve an improved low cross-polarization performance. The height of the antenna is 0.146λ_0_ (λ_0_ is the wavelength of lowest frequency). The prototype antenna demonstrates a measured impedance bandwidth of 93.5% (2.7–7.44 GHz), a 3-dB-gain bandwidth of 77% (2.7–6.1 GHz), and a maximum gain of 10.5 dBi at 4.5 GHz.

## Introduction

With the great development in wireless communication systems, demanding for antennas with light weight, high performance, low cost and ease manufacture, printed antenna is widely used in various systems. Moreover, in order to obtain good pattern performance, the radiation part of many classical antennas also retain the characteristics of symmetry when they are printed on a substrate, such as plane dipole antenna, plane Yagi antenna, Archimedes spiral antenna. However, coaxial line which is most wildly used is an unbalanced waveguide, so balun has a significant role in the balanced-fed-antenna design. Many unbalanced-balanced transform structures have been proposed in the published literature, microstrip to parallel strip lines, CPW transform to parallel strip line, microstrip line coupled to slot line and so on. Especially, microstrip line transform to parallel strip lines is the most widely used structure, for example, some wide-band unidirectional cavity-backed folded triangular bowtie antenna, folded sectorial bowtie antenna or other symmetrical radiation structure are fed by a parallel strip line via a transition from a microstrip line [1–3]. A microstrip gradient line is also used to transform the unbalanced coaxial cable to the balanced Nojima square origami conical spiral structure and realize the impedance transformation [4]. This kind of microstrip balun always is a long structure relatively, because it is necessary for the impedance transformation. A quasi-Yagi antenna fed by coplanar waveguide is presented in [5], however, due to the unbalanced feed for the driver dipole of the quasi-Yagi antenna, deterioration of electrical performance is inevitable, especially the deterioration of its radiation pattern. Therefore, more article adopted the structure of microstrip line coupled to slot line as the balun of the plane quasi-Yagi antenna or Vivaldi antenna [6–8]. This method of feeding can achieve a wider impedance bandwidth on the one hand and can guarantee the symmetry of the radiation pattern on the other hand. There are also some articles adopt the phase-shifting network to achieve the purpose of balanced differentially feed [9] [10]. But the length of the transmission line due to the 180 ° phase shift will vary with frequency, so this kind of feeder network is very difficult to achieve a wide bandwidth.

A wide-band low cross polarization antenna with stable radiation patterns is proposed in this paper. The feed structure of the proposed antenna are two pairs of parallel transmission lines via a transition from a slot line which is coupled by a microstrip line. This structure of microstrip line coupled to the slot line not only completes the conversion of unbalanced feed to balanced feed, but also achieve the role of power divider. Because the radiation part of the proposed antenna is fed by symmetrical parallel transmission lines, the antenna can achieve an improved low cross-polarization performance. The parallel transmission line will not change the inductance of the input impedance, and parallel transmission line is a kind of a naturally balanced transmission line which can match with slot line and radiation patch perfectly. Measurements demonstrate that the impedance bandwidth of the proposed antenna can be extended to 93.2% (2.7–7.44 GHz) and the maximum gain is 10.5 dBi with low cross polarization of 17.5 dB within the whole 3-dB-gain bandwidth (2.7–6.1 GHz).

## Antenna design

### The electrical principle of balun proposed

**Fig 1:**
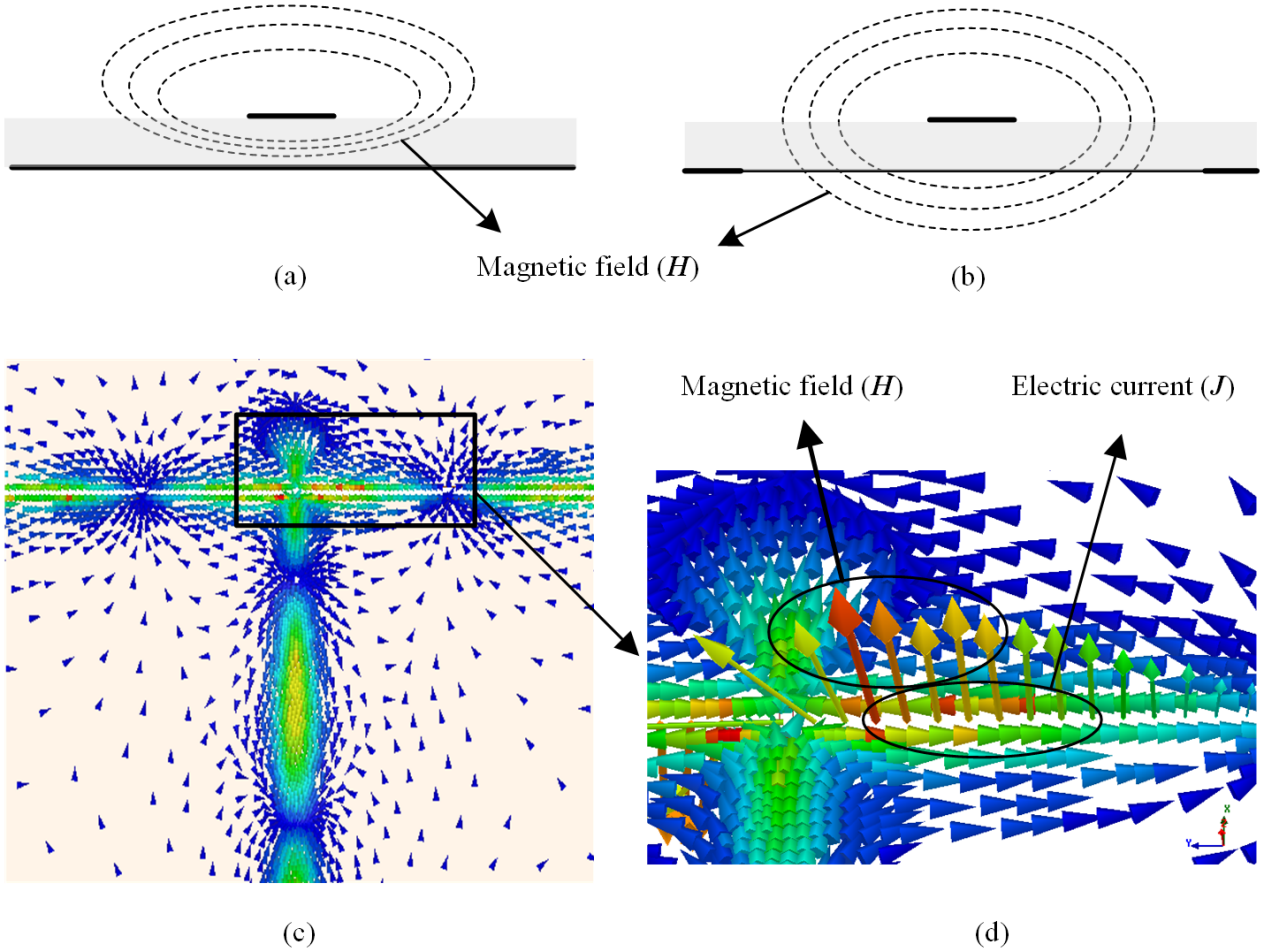
The current and magnetic field distribution of the balun. (a) microstrip line, and (b) the position microstrip line coupling to slot line, (c) current distribution of the balun, and (d) the detail current and magnetic field distribution of the slot line.

A simple diagram about the structure of microstrip line coupling to the slot line, which can achieve the function of transforming the unbalanced feeding to the balanced feeding and a 1:2 power divider is showed in Fig 1. we know that the magnetic fields are around the strip of the microstrip line as shown in Fig 1(a), and all magnetic fields are distributed above the ground. However, the state is different in the position where microstrip line crosses the slot line. As shown in Fig 1(b), due to the existence of the slot, the magnetic field will pass though the ground plane via the slot. It will stimulate two completely reverse current on the two sides of the slot. The simulated current distribution of the balun is shown in Fig 1(c) and 1(d). it can be noticed that currents on the two sides of the slot are with equal amplitude and opposite phase. Hence the transform of the unbalanced feeding to the balanced feeding is achieved. Furthermore, because a complete magnetic induction line will pass through the slot plane twice which locate on the two sides of the wire, thus a 1:2 power divider is formed.

### Antenna structure

**Fig 2:**
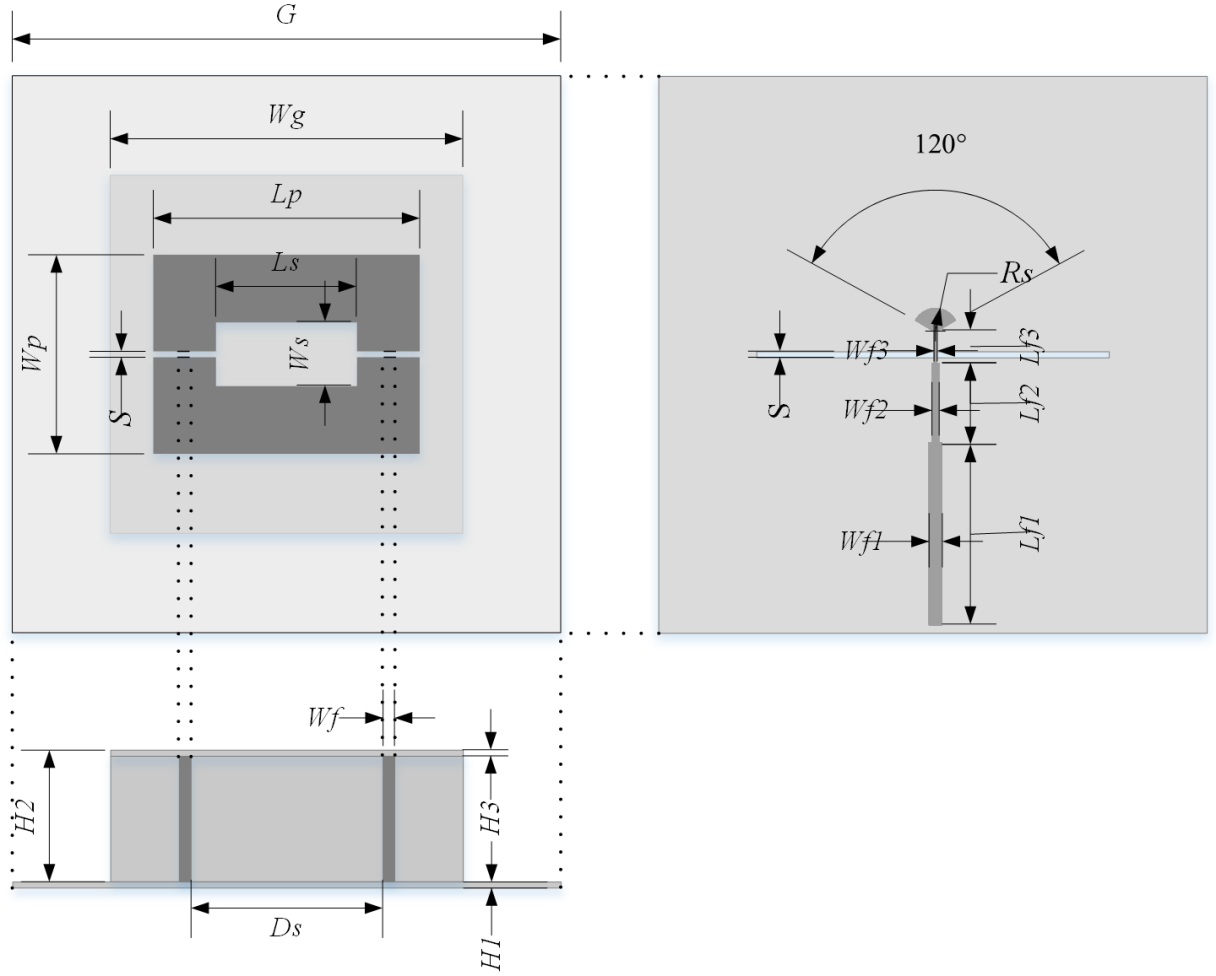
Geometry of the proposed antenna. (top view, bottom view, front view).

The geometric structure of the proposed antenna includes three parts as shown in Fig 2 and details of the parameters are shown in Table 1. The antenna is designed on Rogers RO4350B substrates with a thickness of 0.762 mm, dielectric constant ε_r_ = 3.48 and loss tangent tan δ = 0.004. The total size of the proposed antenna is 70×70×16.26 mm^3^. The antenna consists of three substrates, on which printed a microstrip line to slot line transition feed, two parallel transmission lines and a radiation patch with a rectangular aperture and two feed port, respectively.

**Table 1:** Physical dimensions of the experimental antenna.

Symbol	Quantity	Symbol	Quantity
*G*	70 *mm*	*Wf3*	0.32 *mm*
*Wg*	45 *mm*	*Rs*	3 *mm*
*Lp*	34 *mm*	*H1*	0.762 *mm*
*Wp*	25 *mm*	*H2*	15.5 *mm*
*Ls*	18 *mm*	*H3*	0.762 *mm*
*Ws*	8 *mm*	*Ds*	24.5 *mm*
*S*	0.8 *mm*	*Lf1*	22.6*mm*
*Wf*	1.5 *mm*	*Lf2*	10*mm*
*Wf1*	1.66 *mm*	*Lf3*	4.2*mm*
*Wf2*	0.9 *mm*		

The microstrip transmission line with three-step width decreasing microstrip line is printed on the bottom layer of the bottom substrate and a slot line is printed on the top layer of the bottom substrate which is cross with the microstrip line. The impendence of the finest microstrip line is 120 Ω which is approximate to the characteristic impedance of the slot line. Both the multi-order transformation of the microstrip line and the microstrip sector stub at the end of the microstrip line are inserted in the transition to improve the impedance transformation. On the middle substrate which is perpendicular to the bottom substrate and the top substrate, two pairs of parallel transmission line are printed to connect the slot line and the radiation patch. Especially, the two branches of the both parallel transmission lines are fabricated on two sides of the slot line respectively. Two slots and a rectangular aperture divide the radiation patch into two bent dipoles which fed by the two pairs of parallel lines respectively.

## Computed and measured results

**Fig 3:**
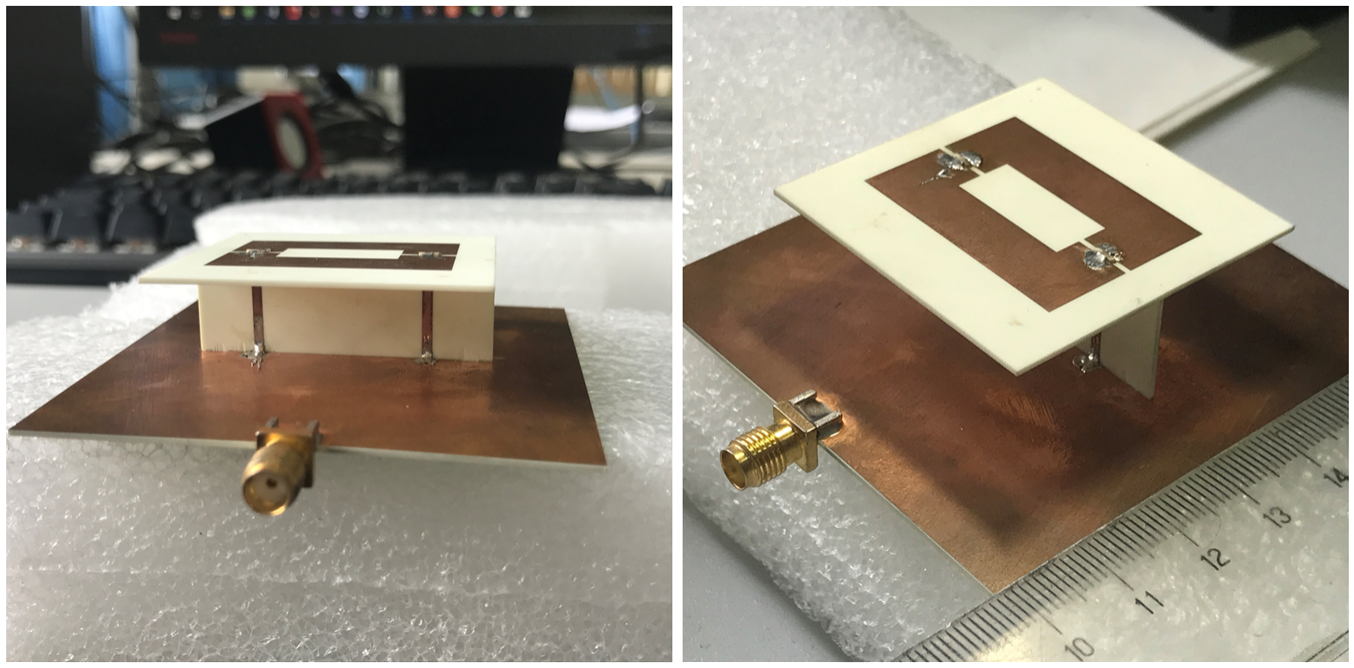
Photograph of the fabricated antenna.

To verify the performance of the antenna design, a prototype according to Fig 2 is fabricated and tested. A 50-Ω SMA connector is fabricated to the microstrip line to stimulate the prototype. The photographs of the fabricated antenna are shown in Fig 3. How to stitch the antenna is shown by the three views correctly in Fig 3 as well.

### Reflection coefficient and gain

**Fig 4:**
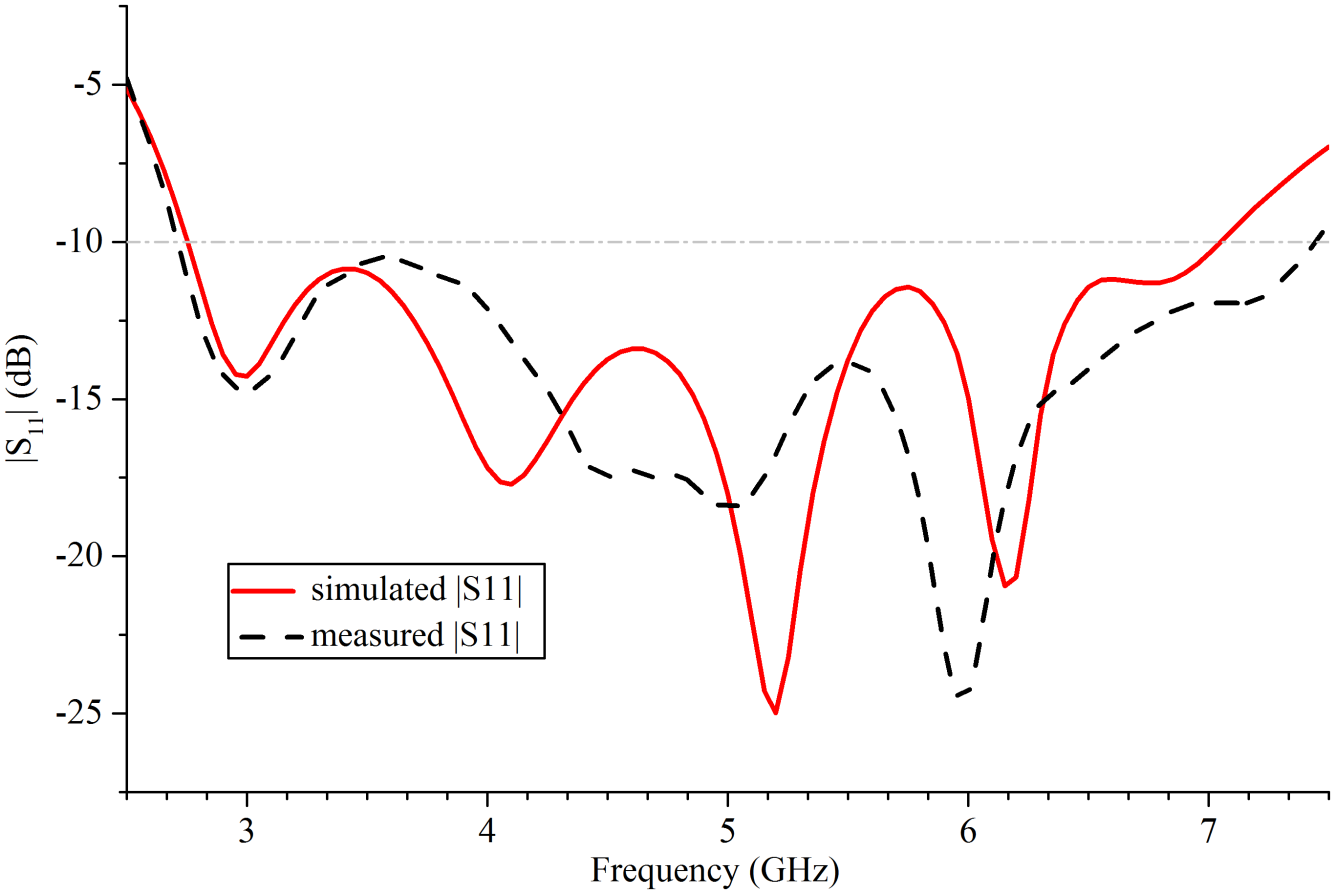
Reflection coefficient of simulation and measurement.

**Fig 5:**
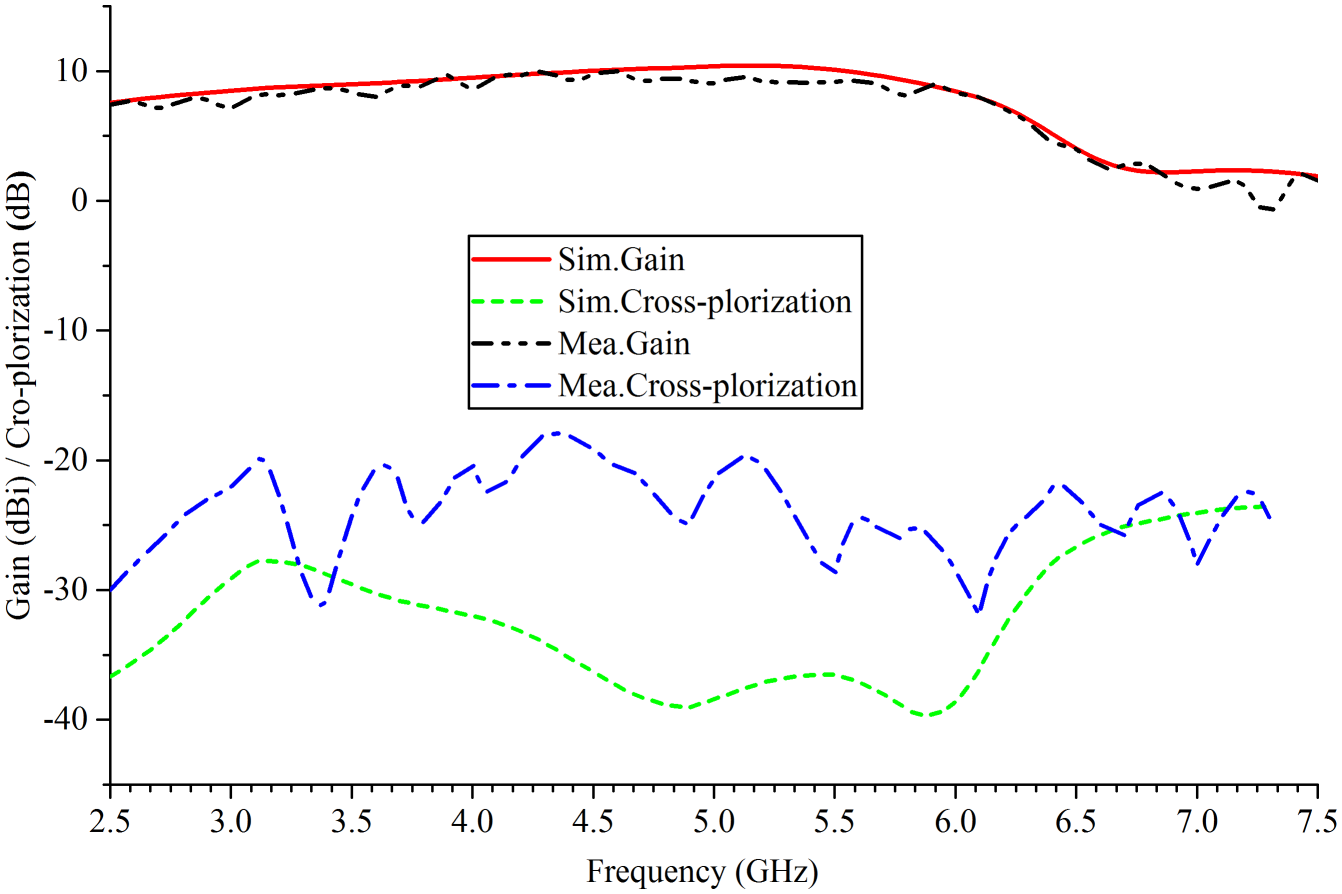
Antenna gain and cross-polarization of simulation and measurement.

Fig 4 demonstrates the simulated and measured reflection coefficients of the fabricated antenna. An acceptable agreement is between the measured and the simulated results. The proposed antenna reaches a 93.2% measured bandwidth ranging from 2.7 to 7.44 GHz at which the reflection coefficient satisfies the |S11| < -10 dB rule. The measured gain and the cross-polarization variating with frequency of the antenna are plotted in Fig 5. The maximum gain is 10.5 dBi at 4.5GHz, and in the band between 2.7 and 6.1 GHz, the fluctuation of measured gain is less than 3 dB. When the frequency exceeds 4.5 GHz, the gain of the proposed antenna will decrease gradually with the increase of frequency.

### Radiation patterns

**Fig 6:**
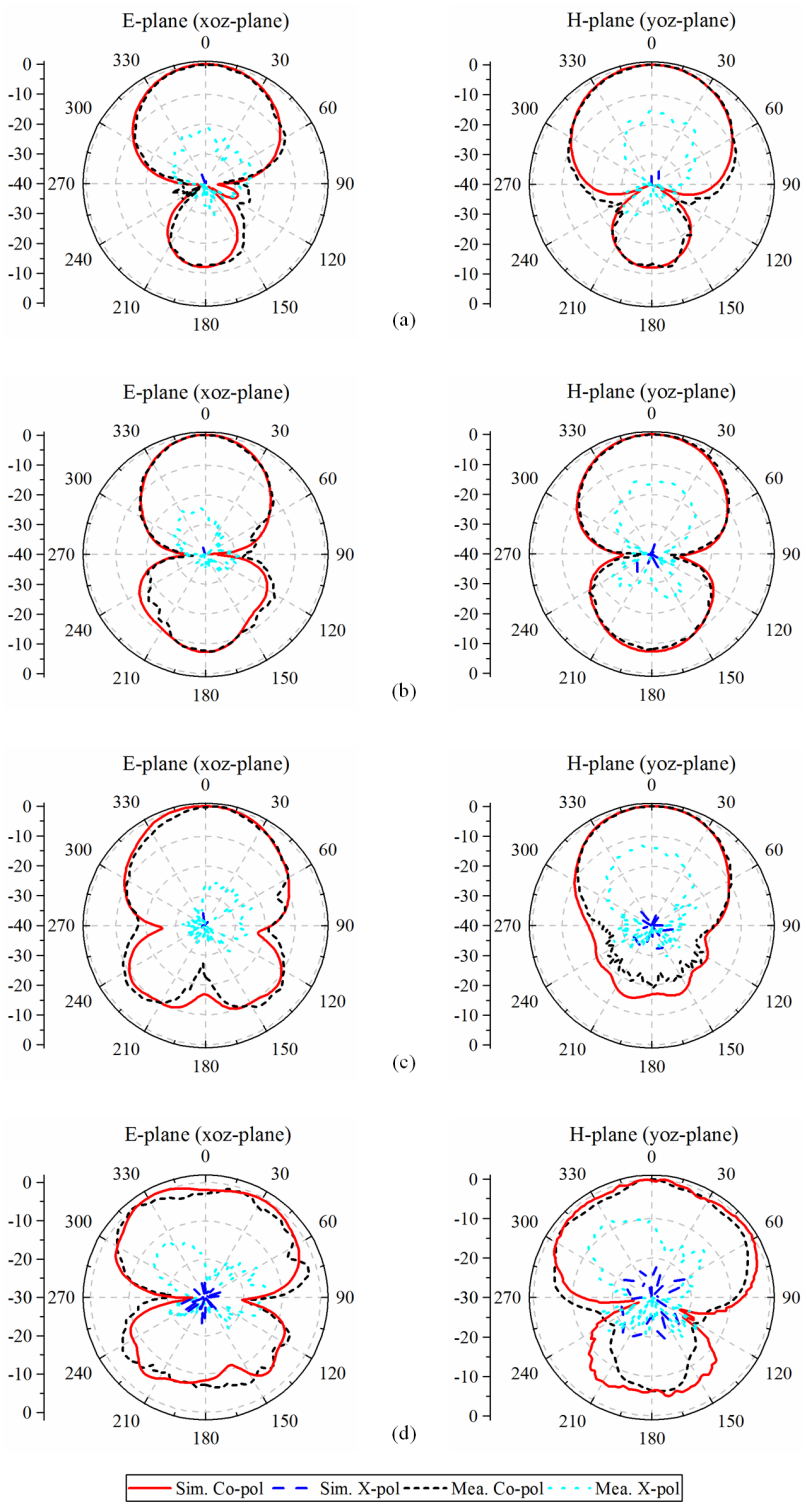
Simulated and measured radiation patterns of the fabricated antenna. (a) 3 GHz; (b) 4.5 GHz; (c) 6 GHz; (d) 7 GHz.

The far-field radiation patterns of the fabricated antenna are measured to verify the radiation performance of the proposed antenna. The simulated and measured far-field radiation patterns in E-plane (*xoz*-plane) and H-plane (*yoz*-plane) at 3, 4.5, 6, and 7 GHz are presented respectively in Fig 6. From it, a good consistency was observed between the computed and measured co-polar. As the figure revealed, for the E-plane and H-plane, the proposed antenna has a substantially equivalent beamwidth. Because the proposed antenna consists of perfectly balanced-fed, a high polarization isolation of more than 27.5 dB is obtained by simulation and the measured polarization isolation is higher than 17.5 dB within the 3-dB-gain bandwidth. The Cross-polarization parameters exist a large gap between the simulation and test results, which is due to double-ridge horn is selected as the transmitting antenna to simplified testing, and did not choose the open waveguide with a higher polarization purity as a transmitting antenna. In addition, the antenna, unfortunately, has relatively high back radiation level, which is due to the existence of the position where the impedance changes intensely (e.g. the end of the slot) and the slot plays a tiny role for radiation inevitably at the same time as a balun. The back radiation levels can be suppressed by adding a back cavity structure. However, the back cavity and the antenna reflector will form a substantially closed resonant cavity which exist a resonance frequency in the operating frequency band. For the performance of the proposed antenna, the resonance will cause an increase in return loss and a decrease in gain at the resonance frequency. Therefore, a lower back radiation level can be achieved with a drawback of unstable performance in the broad operating frequency band.

### Comparisons

Since there is no multi-layer three-dimensional antenna that utilize differential performance of the current on two sides of the slot line. we choose the plane Yagi antennas [7][8] which driver dipole is fed by plane parallel line stretched from slot line to compare. This kind of plane Yagi antennas have a similar principle with the proposed antenna. Generally, the proposed antenna has a higher gain than the plane Yagi antennas proposed in [7][8]. Gains of these plane Yagi antennas are 4–7 dBi universally and the proposed antenna in this paper has a gain advantage about 3 dB at the corresponding frequency. Comparing to the balun achieved by microstrip gradient line [1–4], the Antenna using the balun proposed in this paper can lower the height and achieve an improved low cross-polarization performance. Comparing to the balun used in [9][10], the balun used in this paper is simpler and can obtain a wider impedance bandwidth.

## Conclusion

A wide-band low cross polarization antenna with stable radiation patterns is proposed in this paper. A radiation patch with a rectangle aperture is fed by two pairs of parallel transmission line via a transition from a slot line which is coupled by a three-step width decreasing microstrip line. Because the radiation patch of the antenna is fed by symmetrical structure, the antenna can achieve an improved low cross-polarization performance. Measurements show that the impedance bandwidth can be extended to 93.2% (2.71–7.44 GHz) and 3-dB-gain bandwidth is 75% (2.7–6.1 GHz), and the maximum gain is 10.5 dBi with low cross polarization of 17.5 dB within the whole 3-dB-gain bandwidth.
